# Complete Genome of *Salmonella enterica* Serovar Enteritidis Myophage Marshall

**DOI:** 10.1128/genomeA.00867-13

**Published:** 2013-12-05

**Authors:** Adrian J. Luna, Thammajun L. Wood, Karthik R. Chamakura, Gabriel F. Kuty Everett

**Affiliations:** Center for Phage Technology, Texas A&M University, College Station, Texas, USAa; Department of Biochemistry and Molecular Biology, Pennsylvania State University, University Park, Pennsylvania, USAb

## Abstract

*Salmonella enterica* serovar Enteritidis is a food-borne pathogen that causes salmonellosis in the United States. Bacteriophages are emerging as viable biocontrol agents against this pathogen. Here, we present the complete annotated genome sequence of *Salmonella* Enteritidis T4-like myophage Marshall, which has potential as a phage therapy agent.

## GENOME ANNOUNCEMENT

*Salmonella enterica* serovar Enteritidis infections have been growing in number worldwide since the 1970s and have become the leading cause of salmonellosis illness in humans (1). The *S.* Enteritidis pandemic is largely attributed to the pathogen's unique adaptation for egg contamination (2). The increase in infections makes it necessary to develop a biocontrol method that can be implemented in hen houses. Bacteriophages are ideally suited for this as they can be used in place of antibiotics, which may have unintended downstream effects such as resistance.

Here, we report the sequencing and annotation of *S.* Enteritidis phage Marshall, which was isolated from a sewage sample collected in College Station, TX. Phage DNA was sequenced using 454 pyrosequencing at the Emory GRA Genome Center (Emory University, Atlanta, GA). Trimmed FLX Titanium reads were assembled to a single contig at 41.35-fold coverage using the Newbler assembler, version 2.5.3 (454 Life Sciences), at default settings. Contigs were confirmed to be complete by PCR. Genes were predicted using GeneMarkS (3), and gene predictions were corrected using software tools available on the Center for Phage Technology (CPT) portal (https://cpt.tamu.edu/cpt-software/portal/). Transmission electron microscopy was performed at the Microscopy and Imaging Center at Texas A&M University.

Marshall is a myophage with a genome size of 156,338 bp. It has a GC content of 45.6%, 205 predicted genes, 4 predicted tRNA genes, and a coding density of 92.7%. Genes of all major functional units (DNA replication/recombination/packaging/biosynthesis, morphogenesis, assembly, and lysis) were identified. Marshall is a T4-like phage in the Vi01 family, which includes *Salmonella* phages ΦSH19 and Vi01 and *Shigella* phage SboM-AG3 (4). It is predicted to use a headful DNA packaging strategy based on TerL homology to other large terminases of known packaging strategies. As a T4-like phage, Marshall has a circularly permuted genome that was opened to the *rIIA* gene, by precedent (5). Genome analysis showed that Marshall contains many features similar to those of T4, like *rIIA/B* genes, but has many unique features that make it stand out from other T4-like phages.

A unique feature of Marshall is that it encodes two tail proteins with beta helix/pectin lyase domains as found in phage P22 tail spikes. Multiple tail spike proteins might be an indication of a broad host range similar to that of phage ΦSH19 (4). The pectin lyase domains are hypothesized to be important for biofilm degradation (6). Marshall also contains a putative phosphate starvation-induced, PhoH-like protein which is rarely found in nonmarine phages (7). PhoH has been shown to have ATP binding activity and is predicted to be an ATPase (8). Unlike that of T4, the large terminase subunit of Marshall is interrupted by an intein, as determined by the presence of a Hedgehog/intein (hint) domain (InterPro database entry IPR003587) and an intein splice site (IPR006141) (9).

Six putative homing endonucleases were found. Three of the homing endonucleases contain an HNH domain, and three are from the GIY-YIG family.

### Nucleotide sequence accession number.

The genome sequence of phage Marshall was contributed to GenBank under accession number KF669653.
